# *methylEZ*: a graphical user interface for differential methylation analysis in bisulfite sequencing data

**DOI:** 10.7717/peerj.21795

**Published:** 2026-11-04

**Authors:** Alejandra Rodriguez-Sosa, Sudipto Das

**Affiliations:** Epigenetics of Gastrointestinal Diseases Research Group, School of Pharmacy and Biomolecular Sciences, Royal College of Surgeons, Dublin, Ireland

**Keywords:** DNA methylation, Bisulfite sequencing (BS-seq), Graphical user interface (GUI), Nextflow (nf-core/methylseq), Reproducible workflows, Quality control, MethylKit, Template generator, Picardtools

## Abstract

**Background:**

High-throughput bisulfite sequencing (BS-seq), including whole-genome bisulfite sequencing (WGBS) and reduced representation bisulfite sequencing (RRBS), provides base-resolution DNA methylation. Despite mature command-line (CLI) workflows for alignment, methylation calling and downstream analysis, many researchers without CLI experience face steep setup and reproducibility barriers. Existing graphic user interfaces (GUIs) often target array data or late-stage visualization and rarely assist with containerized, sequencing-oriented workflows that must run on high-performance computing (HPC) cluster or local workstations.

**Methods:**

We present *methylEZ*, an open-source, cross-platform, Python/Tkinter GUI that streamlines preparation of BS-seq analyses while preserving transparent, script-first execution. The application is organized in three modules: (1) preprocessing: FAST-All with Quality (FASTQ) selection, read-pairing, and generation of validated samplesheets and ready-to-run commands for the nf-core/methylseq pipeline; (2) quality control assistance:automation of common asset preparation (reference indexing/dictionaries, Browser Extensible Data (BED) to Picard interval_list conversion, Binary Alignment Map (BAM) sorting/indexing) and batch scripts to drive Picard tools (*e.g*., CollectHsMetrics for capture designs) and samtools; and (3) downstream analysis templating: export of a parameterizable methylKit R script supporting coverage filters, differential methylation (DM) detection (with optional tiling), basic plots and annotation. All artifacts (CLI strings, configs and scripts) are saved for inspection, version control and reuse.

**Results:**

We validated *methylEZ* on in-house methyl-capture BS-seq data and on publicly available BS-seq data. The GUI correctly produced nf-core/methylseq samplesheets and Nextflow commands, and the quality control (QC) module batched reference/BAM preparations and coverage summaries *via* scripted Picard/samtools steps, reducing repetitive CLI interaction. The exported methylKit templates executed without error and generated interpretable differential methylation outputs. *methylEZ* runs on macOS, Windows and Linux; it is installable *via* pip from GitHub and requires Python 3; Tkinter is bundled on Windows/macOS and typically available *via* the Tk package on Linux.

**Conclusions:**

*methylEZ* lowers the entry barrier to reproducible BS-seq by moving error-prone configuration and QC setup into a guided GUI while keeping execution explicit and auditable through exported commands and scripts. The tool complements web platforms (*e.g*., Galaxy) by emphasizing offline control and compatibility with community workflows (nf-core/methylseq) and statistical packages (methylKit) on capable infrastructure. Planned extensions include in-GUI visualization (*e.g*., PCA, coverage histograms, volcano plots) and support for alternative callers and statistical frameworks. *methylEZ* is released under GPL-3.0.

## Introduction

DNA methylation is an epigenetic mechanism that involves the transfer of a methyl group to the C5 position of cytosine (C), resulting in 5-methylcytosine (5mC). Methylation typically occurs on cytosines preceding a guanine (G) or cytosine–phosphate–guanine (CpG) sites (Moore, Le & Fan, 2012). In mammalian somatic genomes, a 70–80% of the CpG dinucleotides are methylated, except for CpG islands. These are regions rich in guanine–cytosine (GC) commonly located at gene promoters, and frequently found unmethylated in germ cells, early embryos and most somatic tissues (Bird, 2002; Li & Zhang, 2014). Historically, bisulfite sequencing (BS-seq) has been considered the gold standard for determining DNA methylation at single-base resolution (Ehrich et al., 2007; Gouil & Keniry, 2019) and has been used widely to develop detailed DNA methylation profiles across different species as well as in humans specifically disease and healthy states.

In recent years, emerging alternatives have revolutionized methylation analysis. Enzymatic methyl-seq (EM-seq) employs enzymatic conversion to reduce DNA damage compared to bisulfite treatment, which improves library yields, reduces polymerase chain reaction (PCR) duplication, and enables the analysis of low-input samples, all while detecting 5mC and 5hmC simultaneously (Vaisvila et al., 2021). Furthermore, long-read technologies such as Oxford Nanopore Technologies (ONT) directly detect DNA methylation without chemical treatment, facilitating the analysis of highly methylated regions and repetitive elements (Liu et al., 2021). Similarly, PacBio HiFi long-read sequencing (15–25 kb) provides high-confidence methylation detection in complex genomic regions (Wang et al., 2025). Despite these advances, BS-seq remains highly relevant in the field (Seiler Vellame et al., 2021; Gong et al., 2022). It continues to offer single-base resolution genome-wide coverage at a lower cost than long-read methods, benefits from well-established protocols and extensive published datasets, and remains better characterized for clinical and diagnostic use, making it suitable for most research applications requiring comprehensive CpG methylation profiling.

DNA methylation plays a key role in multiple cellular processes, like genomic imprinting, X-chromosome inactivation and regulation of gene expression (Jin & Liu, 2018). Disruption of this mechanism has been vastly associated with the development of pathologies such as cancer, with aberrations in DNA methylation having been observed in different cancer types like colon, breast, liver, and many others (Jin & Liu, 2018). In autoimmune diseases, such as rheumatoid arthritis, DNA methylation alteration has been correlated with genetic susceptibility (Liu et al., 2013). Similarly, multiple sclerosis, and changes in DNA methylation have also been correlated with genetic susceptibility (Meng et al., 2017). Beyond immune related diseases, the role of DNA methylation is crucial as well in developmental neurological disorders, metabolic disorders and aging, remaining a ubiquitous process in human biology (Dayeh et al., 2014; Iossifov et al., 2014; Jin & Liu, 2018).

During the bisulfite conversion process, key step of BS-seq, the methylated cytosines remain methylated, and the unmethylated ones are replaced by uracil (U). Running PCR amplification, the U nucleotides are replaced by thymine (T) while the methylated Cs remain unchanged. This conversion C > T and C > C is analysed by available bioinformatic tools to quantify DNA methylation levels (Frommer et al., 1992). Although multiple computational tools, such as Bismark (Krueger & Andrews, 2011), BS-Seeker2 (Guo et al., 2013), methylKit (Akalin et al., 2012), and RnBeads (Assenov et al., 2014; Müller et al., 2019) facilitate the processing and analysis of BS-seq data (Wreczycka et al., 2017), their complexity and the statistical expertise required to use them effectively can hinder their adoption by lab-based scientists. An accessible platform facilitating this workflow is therefore an unmet need.

Multiple computational tools have attempted to bridge this gap addressing complexities of BS-seq data analysis in the past years. For instance, tools such as *Methyl-C analyzer* (Lu et al., 2023) provide a robust downstream analysis and visualization platform for methylation data across species. However, despite incorporating a graphic user interface (GUI), it is still largely command-line and script-driven, with some visualization features. In addition, as it focuses on post-alignment analysis, it therefore needs pre-processed sequencing data as input and thus requiring some prior bioinformatics experience and scripting knowledge. Another tool that focuses on streamlining BS-seq analysis is msPIPE (Kim et al., 2022), which presents a containerised approach with Docker, and including every step from alignment to publishable-quality plots, but it operates fully through scripting and command-line (CLI) commands. Since it has no desktop GUI option, it serves as a great example for a very robust workflow that can be harder to use by wet-lab users. In contrast, while *MethylR*, (Volpe & Das, 2023) simplifies the whole downstream differential methylation analysis, it is exclusively designed for Illumina Infinium array data (both 450k and EPIC BeadChips) and does not handle sequencing data. Similarly, *ShinyÉPICo* and *MethGET* (Morante-Palacios & Ballestar, 2021; Teng et al., 2020) provide intuitive GUI-based interfaces yet are also designed for microarray methylation datasets rather than BS-seq data. Beyond individual tools, popular workflow environments such as *GATK* toolkit, *ENCODE* or *Galaxy* (Van der Auwera & O'Connor, 2020; The ENCODE Project Consortium, 2012; The Galaxy Community, 2024) have facilitated the integration and automation of complex analyses. Although these platforms provide a non-negligible automation and contribute towards the standardization of the process, they are still constrained by multiple aspects. The advantage of peer-maintained frameworks like nf-core and its suite of pipelines is their containerisation, regular maintenance and version control (Ewels et al., 2020). This makes these pipelines strong and reproducible, suitable to be used across different computational environments and aligned with the goals of tools like *methylEZ*.

On the one hand, their exclusively web-based environments limit flexibility for local data processing. This makes it complicated to integrate a workflow with a high-performance computing (HPC) clustera, and it also raises concerns regarding data privacy. The processing capacity of some of these tools is another aspect that must be considered as a limiting factor, since resource allocation can greatly vary amongst platforms, and might be a source of unreliability. Finally, as most of these pipelines closed environments, they are susceptible to deprecation when updates are not integrated, potentially impacting functionality and reproducibility, and in most cases, the maintenance of the tool has a high dependency on the developers, which is not always granted.

A potential solution to overcome limitation of these current pipelines, would involve development of a comprehensive environment capable of taking data from input to output, requiring minimal user programmatic intervention, while allowing flexibility and upgradability.

To overcome this challenge, *methylEZ* proposes an end-to-end user-friendly GUI, specifically designed to reduce the barrier for BS-seq data analysis and to continue developing reproducible research in the context of bioinformatics, with a focus on epigenomics.

## Materials and Methods

### Datasets for validation

*Dataset 1*: consisted of tumour and matched tumour-free specimens (*n* = 117) processed using the Agilent SureSelect XT HS2 Methyl-Seq protocol. Libraries were sequenced at Novogene (paired end, 150 bp) in two runs. The final analytic subset included 45 libraries (36 non-metastatic, nine metastatic), used for downstream differential methylation analysis.

*Dataset 2*: consisted of cell line derived samples (*n* = 51) prepared using a custom hybridization-based capture protocol for methylation profiling, sequenced at Novogene (paired end).

*Dataset 3*: consisted of reduced representation (RRBS) samples (*n* = 26) Genomic DNA spiked with lambda DNA was digested using methylation-insensitive restriction enzyme MspI, followed by end repair and dA-tailing. Fully methylated adapters were used for ligation. After size selection, DNA fragments were bisulfite converted and PCR amplified. Libraries were sequenced at Novogene (paired-end, 150 bp, Illumina NovaSeq) in a single run, with quality control thresholds applied for on-target alignment (>10%), mean target coverage (>10×), and duplication rates (<75%).

*Dataset 4 (Public Benchmark):* Consisted of publicly available enhanced reduced representation bisulfite sequencing (ERRBS) data from the Gene Expression Omnibus (GEO accession: GSE93507; Kotini et al., 2017). Coverage files from six samples, two untreated controls and four 5-AzaC-treated haematopoietic progenitor cells derived from a low-risk multidimensional scaling (MDS) iPSC line (MDS-1.12), were processed using the *methylEZ* methylKit template. This dataset was utilised to provide an independent, reproducible validation of the tool’s differential methylation analysis functionality on a well-characterised, published epigenomic dataset, with all generated scripts and outputs available for transparency.

*methylEZ* was developed in Python 3 (van Rossum & Drake, 2009) using the built-in Tkinter (tkinter, 2024) library for GUI rendering. The application follows a modular approach, with a very clear separation of user interface components and logic for command generation, file management and pipeline integration. The GUI is packaged as a standalone tool, installable *via* pip, or runnable from the terminal, supporting MacOS, Linux and WindowsOS. The source code is freely available on GitHub under a GPL-3.0 license at the author’s repository https://github.com/AlejRSosa/methylEZ. The logic of the GUI architecture is depicted in Fig. 1.

**Figure 1:**
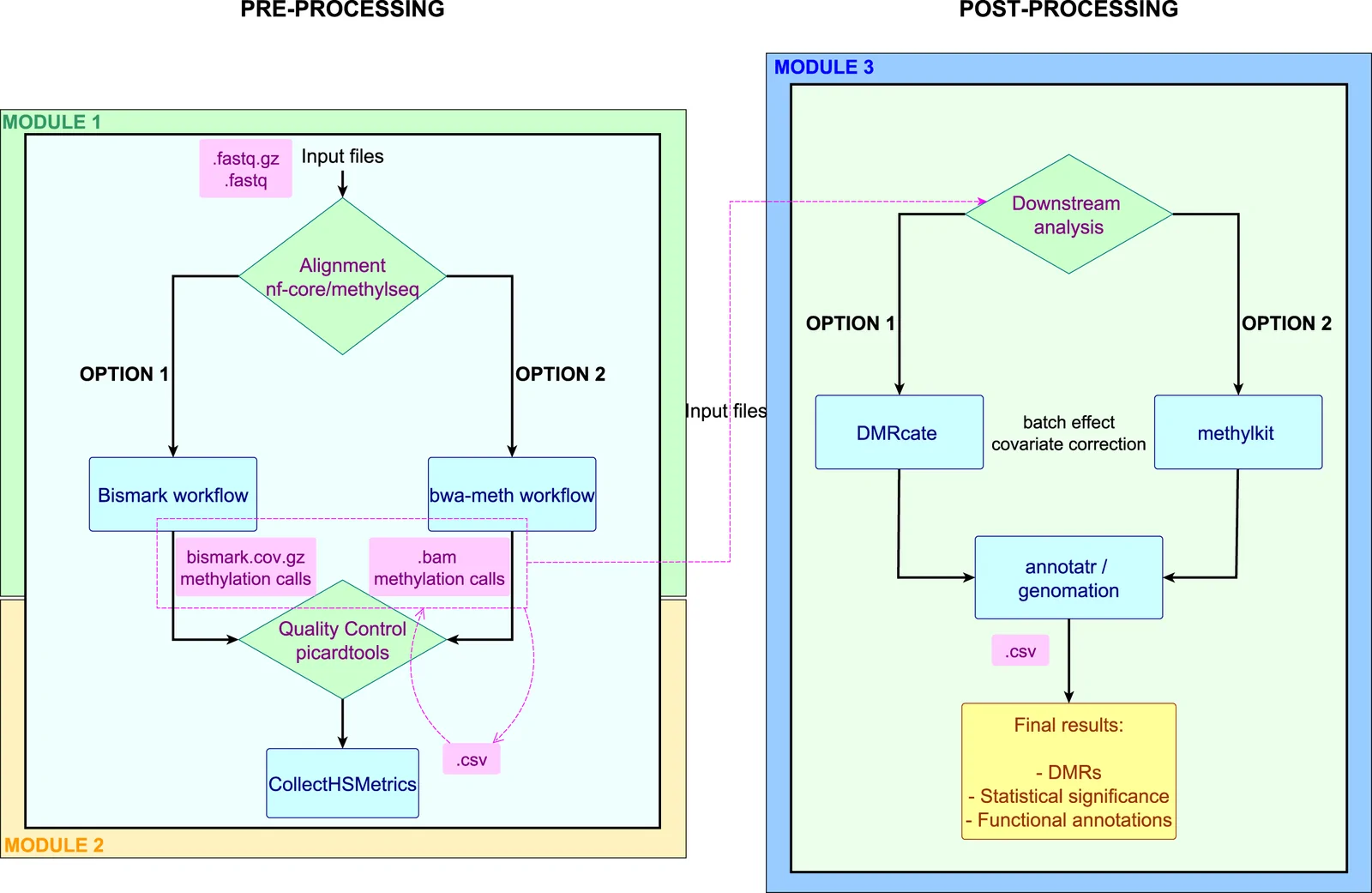
Logic workflow of *methylEZ*. Representation of the three core parts of *methlyEZ*. Module 1 (pre-processing) handles raw sequencing input files (.fastq, .fastq.gz), generating nf-core/methylseq commands and samplesheets for either bwa-meth or bismark workflows [CM1]. In Module 2 there is an [CM2] integration with PicardTools’ CollectHsMetrics for QC on alignment outputs (.bam, .bismark.cov.gz), [CM3] producing tabulated metrics in .csv format [CM4]. This output is then useful to determine which alignment outputs can be used for the differential methylation analysis step. Module 3 (methylation analysis) takes as input the chosen alignment output files (those that pass QC) and generates a methylkit template (.R). Final outputs include differentially methylated regions (DMRs), statistical significance values and functional annotations. Arrows show how the data flow between modules, dashed lines show interactions between the different parts of the pre-processing stage.

Pandas (McKinney, 2010) is utilized for tabular data processing, while Pyperclip (Robertson, 2023) is used to manage clipboard operations for copying commands. The GUI architecture is divided into three core modules that reflect distinct stages of BS-seq analysis: methylseq preparation, CollectHsMetrics (Broad Institute, 2019) setup, and downstream differential methylation script generation using methylKit.

The *methylEZ* system design, which summarises the key inputs, module-level processing steps, decision points and corresponding outputs that action the workflow described in Fig. 2.

**Figure 2:**
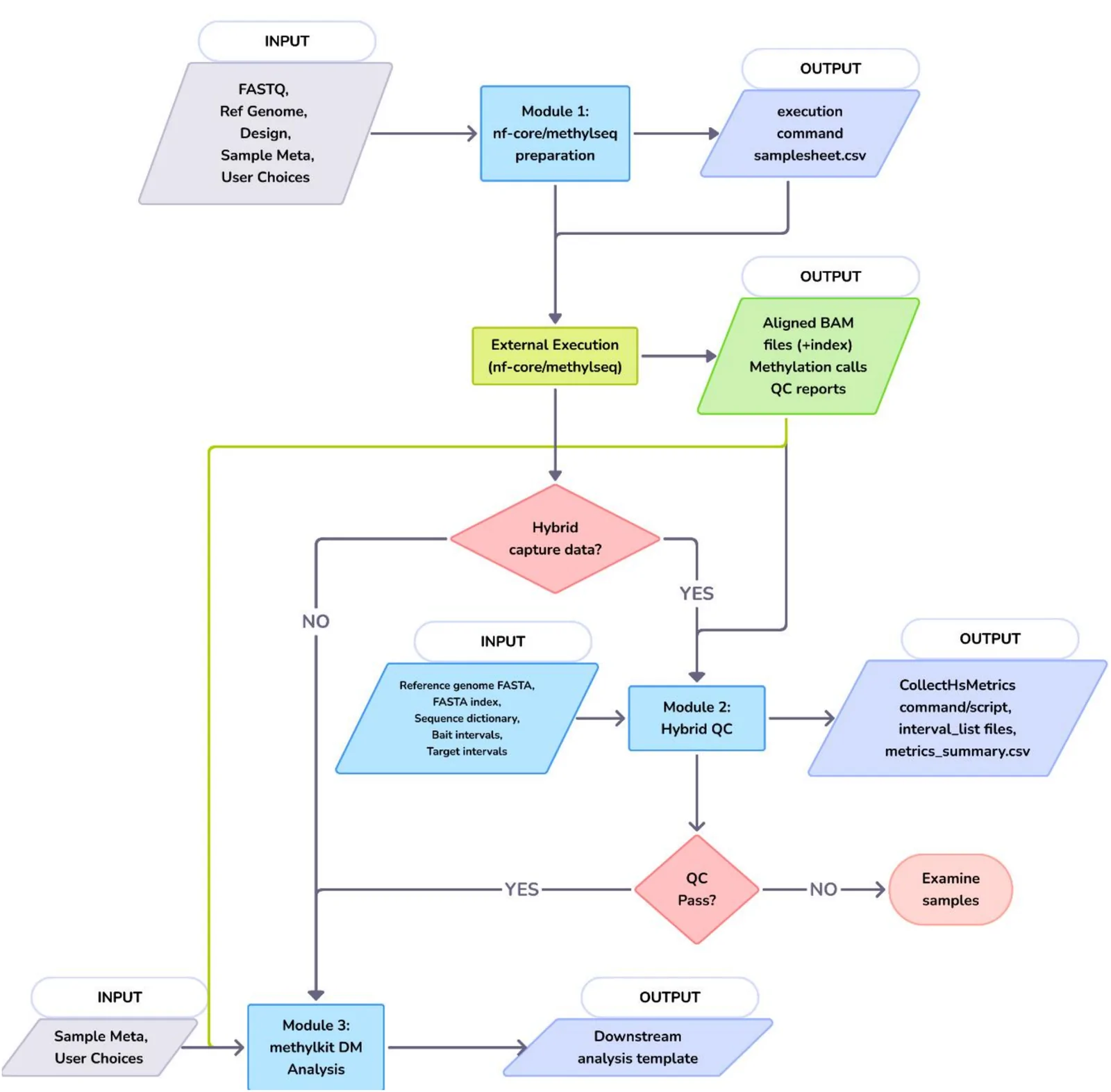
Details of the system interfaces (inputs/outputs) and decision points. System design of *methylEZ* showing the three modules (nf-core/methylseq preparation, optional hybrid-capture QC, and downstream DM analysis), including inputs, decision gates, and generated outputs.

To complement the system-level architecture, Fig. 3 summarises the user-system interactions of *methylEZ*. We highlight the inputs provided by the users, the configurable choices available and which outputs are returned at each stage from the user’s perspective.

**Figure 3:**
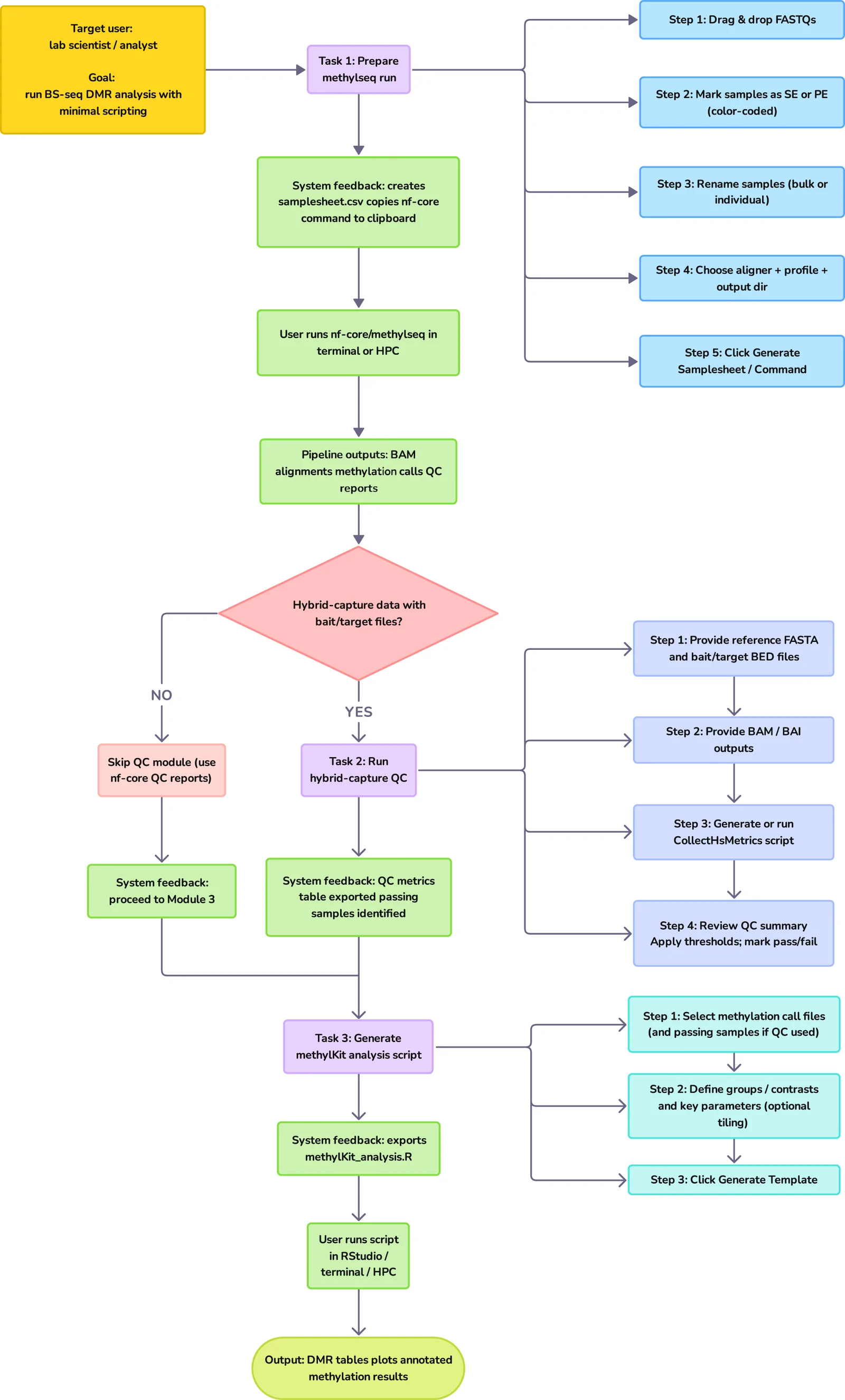
Details of the user interface. The user-interaction schema of *methylEZ* highlights the sequence of tasks to be performed through the GUI and the external execution steps (nf-core pipeline, R DM analysis) that produce the final results.

#### Module 1: methylseq preparation (pre-processing)

The pre-processing module enables users to organize raw sequencing files (.fq, .fastq, *.fastq.gz formats accepted) through a drag-and-drop interface. The files can be imported, reordered and cleared as needed. The first option for the user is to mark the files as single-end or paired-end, following nf-core/methylseq requirements to build a spreadsheet in order to run the pipeline (https://nf-co.re/methylseq/2.6.0/). This status is color-coded, when a sample is single-end it gets highlighted in blue, and if it is paired-end, in yellow. This action is fully reversible by selecting the marked sample and assigning the value “Neutral” from the drop-down menu. Samples who are not marked will not be incorporated into the final spreadsheet.

The option for bulk or individual re-naming is also available. By default, the generated spreadsheet removes the format and keeps only the names of the samples in the sample column, while including the full path of the samples for fastq_1 and fastq_2 columns. However, it is up to the user to modify the spreadsheet once it is built. Once sample marking and naming is selected, the user can select the aligner, bwa-meth or Bismark and the profile, docker or singularity, and specify an output directory. The spreadsheet name by default is “samplesheet.csv” but this can also be modified by the user as required. Three buttons: “Generate Samplesheet”, “Generate Command” and “Generate Both”, allow the user to generate both a spreadsheet with the samples fully ordered and formatted to be used and a command for a standard, unmodified run of nf-core/methylseq which can be copied to clipboard and ran or inserted into an HPC script.

#### Methylation calling and quality control in nf-core/methylseq

Because *methylEZ* serves as a command generator interface for the nf-core/methylseq pipeline, it is crucial to understand the underlying methylation calling and quality control processes. Depending on the user’s selection, alignment is performed using either Bismark (default) or the bwa-meth/MethylDackel workflow. Both aligners handle the reduced sequence complexity of bisulfite-converted reads through specialized alignment algorithms, typically employing a three-letter alphabet or wildcard alignment strategy. At the read level, the pipeline automatically performs quality control *via* FastQC, adapter trimming *via* TrimGalore, and alignment quality filtering using MAPQ thresholds. To mitigate PCR amplification biases stemming from degraded bisulfite-treated DNA, deduplication is performed using Bismark’s deduplication module or Picard MarkDuplicates, depending on the chosen aligner.

For methylation extraction, Bismark extracts methylation calls and reports conversion efficiency statistics directly in its summary reports. Conversely, MethylDackel (used in the bwa-meth workflow) applies default minimum mapping quality (MAPQ) (≥10) and Phred quality (≥5) filtering, with options for coverage thresholds and variant allele fraction filtering (*e.g*., --maxVariantFrac 0.25). The pipeline systematically addresses bisulfite conversion artifacts, where incomplete bisulfite conversion is monitored by calculating conversion rates from non-CpG cytosines (non-CpG cytosine contexts, where H = A, C or T (CHH/CHG) contexts), which should exhibit very low methylation if conversion is complete. Users are advised to inspect Bismark reports or Multiquality control (QC) summaries to verify conversion rates exceed 98–99%. While nf-core/methylseq provides conversion rate statistics using downsampled reads or spike-in controls and automates these essential steps, users should note that certain advanced quality control steps recommended by established methodological standards (*e.g*., coverage thresholds, detection *p*-values for reliable signal, and standard deviation filtering for variable CpGs) are not fully automated.

As a consequence of this, downstream manual filtering is recommended when using the output for differential methylation analysis, such as removing SNP-overlapping probes or applying context-specific coverage-based filtering.

#### Module 2: quality control setup *via* CollectHsMetrics

Many bisulfite sequencing (BS-seq) experiments use RRBS, whole genome (WGBS) or enzymatic methyl-seq, which do not employ capture probes. In those cases, this QC layer is not applicable, since *CollectHsMetrics* cannot be run without the bait/target interval files to calculate enrichment and coverage. This is therefore a QC layer for hybrid-capture users, while reduced representation bisulfite sequencing (RRBS) and whole genome bisulfite sequencing (WGBS) data will get their QC control thanks to the nf-core/methylseq pipeline, which bundles FastQC, MultiQC and Qualimap. To provide a further layer on quality control in hybrid-capture bisulfite workflows, *methylEZ* bundles Picard Tools and provides an interface to run CollectHsMetrics. This tool is used to evaluate the performance of targeted sequencing assays, reporting both basic sequencing metrics and target coverage statistics. This is an important step that allows to assess if the data is suitable for downstream analysis. Given that this QC step is only applicable to hybrid-capture data, *methylEZ* allows the users to completely skip this module and continue straight to Module 3.

CollectHsMetrics requires a specific set of formatted files as input. These files must be the FASTA file of the reference genome of choice, alongside with its index file and sequence dictionary. In addition, it also requires the alignment files as coordinate-sorted BAM files, one per sample along their BAM index files, and finally the target definition files for the bait intervals file and the target intervals files (Table 1). *methylEZ* automates both the preparation and verification of these components, acting as a wrapper to generate and export both the commands and the final files needed to run the tool.

**Table 1:** Commands and tools required to prepare input files for running Picard Tools CollectHsMetrics. The necessary file formats, the corresponding tools and commands used to prepare them, and the resulting output file formats. Preparation steps include indexing the reference genome, generating a sequence dictionary, sorting and converting BED files to Picard-compliant interval lists, and sorting and indexing BAM files.

Input file	Tool + Command required	Output file
Reference genome in FASTA format	Samtools faidx	Index reference sequence in the FASTA format or extract subsequence from indexed reference sequence. (.fai)
	Picard CreateSequenceDictionary	Creates a sequence dictionary (.dict) file from reference sequence.
BED file	Bash scripting	Sorts the BED file before converting to interval format (.interval_list)
	Picard BedToIntervalList	Converts the sorted BED file to a Picard .interval_list
BAM file	Samtools sort	Sort alignments. If sorted output file is to be indexed, the default coordinate sort must be used.
	Samtools index	Index .bai for BAM or .crai for CRAM

In addition, running CollectHsMetrics requires that the reference and sample files are prepared and formatted, which *methylEZ* handles by integrating samtools (Li et al., 2009). The dictionary for the reference genome is created using picard’s CreateSequenceDictionary option. Both bait and target regions preparation, usually provided as .bed files by the sequencing provider or the lab are sorted and cleaned and finally converted to the required *.interval_list file format using Picards’ BedToIntervalList tool. See the following table for input/output requirements. Finally, this second module provides a reusable Python script that runs metrics collection and outputs either a .csv file or the Python parser script with the logic used to summarise Picard metrics across multiple samples, customised with the input and output directories selected through the interface.

The preparation pane (left) and the CollectHsMetrics pane (right) can be used independently. While the user is not required to prepare the FASTA, the BED or the BAM files, however, it is a mandatory requirement that they have the right files ready to be used. Picard commands can be run within the GUI, but the user can also choose instead to generate a template that can then be modified and executed by making it executable with chmod +x <namefile.py> and then running it from a cmd or PowerShell terminal.

#### Module 3: differential methylation template generation

The third module of *methylEZ* addresses the analytical requirements that follow methylation calling, specifically, differential methylation analysis and downstream statistical processing, by assisting users in generating a template script for downstream differential methylation analysis using the package methylkit. Unlike the previous sections, which have the capability of running the commands directly within the GUI and provide both direct outputs and/or customisable templates, in this module the generated script here is a methylkit analysis .R file. While methylkit itself provides a robust suite of functions for methylation analysis, it also requires a proficient level in R scripting. Setting it up is time-consuming and can be convoluted. The interface aims to solve this by providing a set of configurable options that get updated in the generated .R file.

The generated script follows a carefully curated methylKit workflow, including code for object creation, differential methylation testing using logistic regression, optional tiling of genomic regions, and exporting results to tabular formats. The script is structured into clearly labelled sections, allowing for easy modification or integration into a larger analysis pipeline. Users can run the generated script, which incorporates the required packages for the analysis, in RStudio or adapt it for use within notebooks or batch-processing environments.

### Design decisions and parameter defaults

To maintain transparency and prevent a “black box” analytical environment, *methylEZ* relies on several explicit design choices and default parameters. For pre-processing execution, the tool defaults to the *docker* profile to ensure containerized reproducibility. For alignment, the tool defaults to *bismark*, prioritizing a well-established algorithm, though users can toggle to *bwameth* or *bismark*_*hisat*. By default, the interface populates GRCh37 (or hg19) as the reference genome build for the pipeline and downstream analysis, but users are prompted to provide their own specific FASTA files and generated indices for hybrid-capture quality control.

Crucially, *methylEZ* itself does not apply independent quality filtering parameters to the raw data during the pre-processing stage; rather, it passes default parameters to the underlying nf-core/methylseq pipeline, which handles adapter trimming and MAPQ filtering. However, to assist users in downstream differential methylation analysis, the exported methylKit R template applies several standard default thresholds to ensure data reliability: a minimum coverage threshold of 10 reads per CpG, a maximum coverage percentile of 99.9 to remove potential PCR duplication artifacts, and a standard deviation cutoff of 2.0 to filter out CpGs with low variability across samples. For region-level analysis, the template defaults to a tile window size of 500 bp and a step size of 300 bp. All of these parameters are fully exposed in the generated template and the GUI, allowing users to modify them based on experimental requirements. 

## Results

### *methylEZ* functionality results

The software was successfully tested using a series of methylation capture sequencing datasets from different research groups at the Royal College of Surgeons in Ireland (for internal research use only). The datasets employed span colorectal tumour and matched normal tissue, RRBS profiles and cell line derived methylation libraries. The software was tested for automated samplesheet generation, nf-core/methylseq command creation and execution, multi-BAM QC parsing with PicardTools and methylKit script analysis template generation.

To illustrate *methylEZ*’s functionality and performance, we present the results for each module in the order they have been introduced in the methods section. For the evaluation of module 1, pre-processing, *methylEZ* successfully generated valid nf-core/methylseq commands and samplesheets for all test cases, accurately reflecting sample-level metadata and file structures. The generated commands were run on our local HPC and produced the intermediary files and reports consistent with manual pipeline execution. Figure 4 shows the arrangement of panes and different functions of the tool in this section. The second module tested was the QC module running CollectHsMetrics from PicardTools. In Fig. 5 we can observe the interface and the different options, from running commands within the environment to generating scripts. The HsMetrics module successfully processed multiple BAM files by exporting a multi-sample executable script and accompanying parser. Output .csv files included standard Picard metrics such as percent aligned bases, mean target coverage, and fold enrichment.

**Figure 4:**
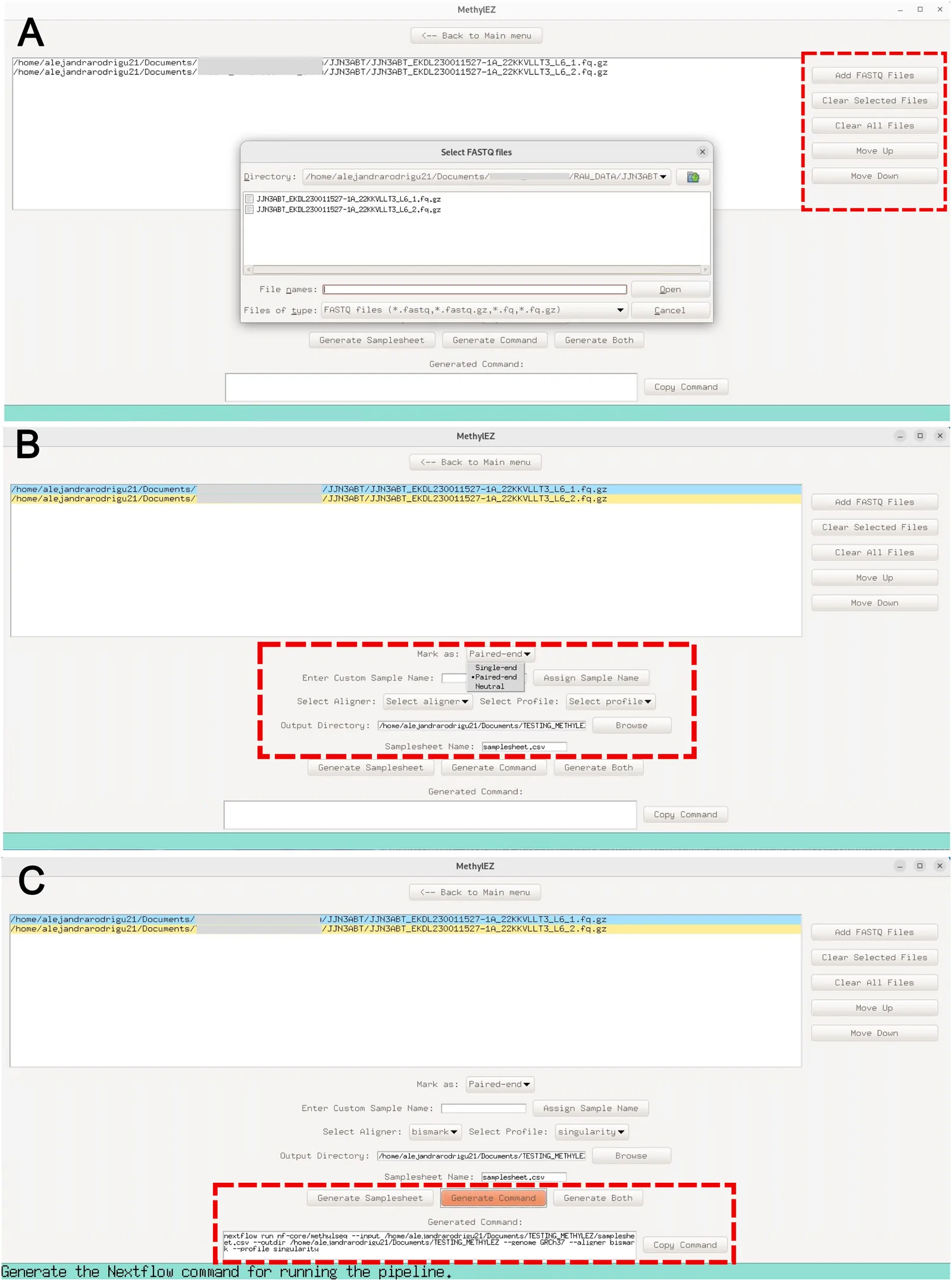
Module 1-Pre-processing interface. (A) Highlights the available commands for adding, removing and rearranging the files within the GUI. (B) Shows different menus that correspond to options of nf-core/methylkit pipeline such as paired-end or single-end read marking, sample name customization, alignment selection, output directory selection *via* browser button and samplesheet name customization tab. In the main panel, two test samples have been marked in different colours to show the functionality of marking as paired-end (blue) or single-end (yellow) (C) Command generated with the test samples by using the Generate Command button. The command can be seen in the pane below and can be copied to clipboard with the Copy Command button or with cursor selection and Ctrl+C shortcut.

**Figure 5:**
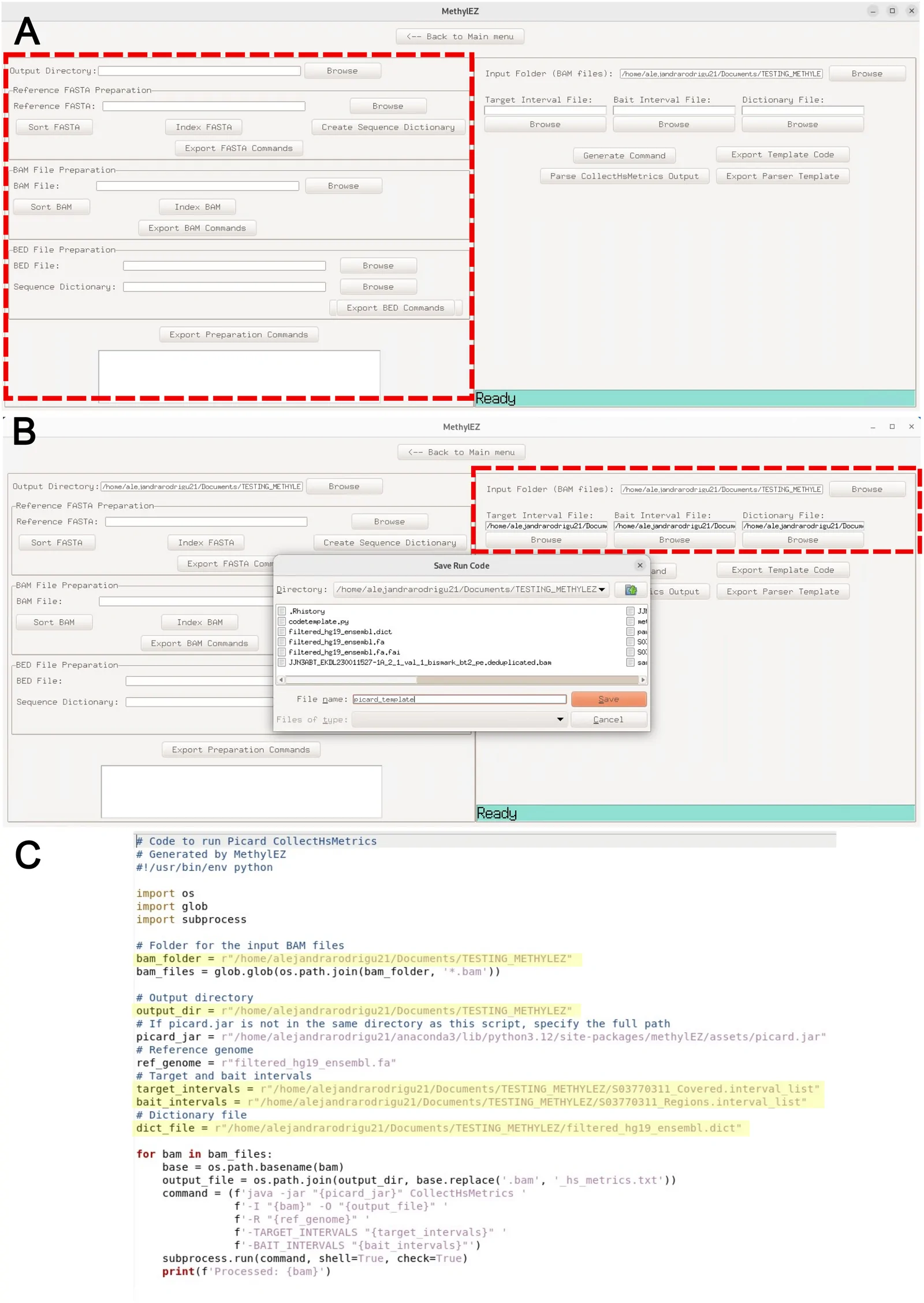
Module 2-CollectHsMetrics interface. The options to generate all the files to run CollectHsMetrics or to directly obtain the template scripts to run CollectHsMetrics if the user already has those files ready. (A) The three sub-sections, Reference FASTA preparation, BAM file preparation and BED file preparation are completely independent of each other. The Export Preparation Commands button does require the use of the other three sub-sections. (B) The final command script generation takes place in this section of the GUI. The output is a script to run the command or a script to run a parser that takes the result of CollectHsMetrics and saves it into an easy to read .csv file.

Finally, the methylKit script generator or third module produced reproducible analysis templates that were compatible with methylKit (v1.24.0) and ran without error. These scripts yielded differentially methylated region (DMR) results and plots (*e.g*., heatmaps, PCA, volcano plots) consistent with expected sample groupings, validating the script logic. A screenshot of the interface of this third module together with an example of the produced output can be seen in Fig. 6.

**Figure 6:**
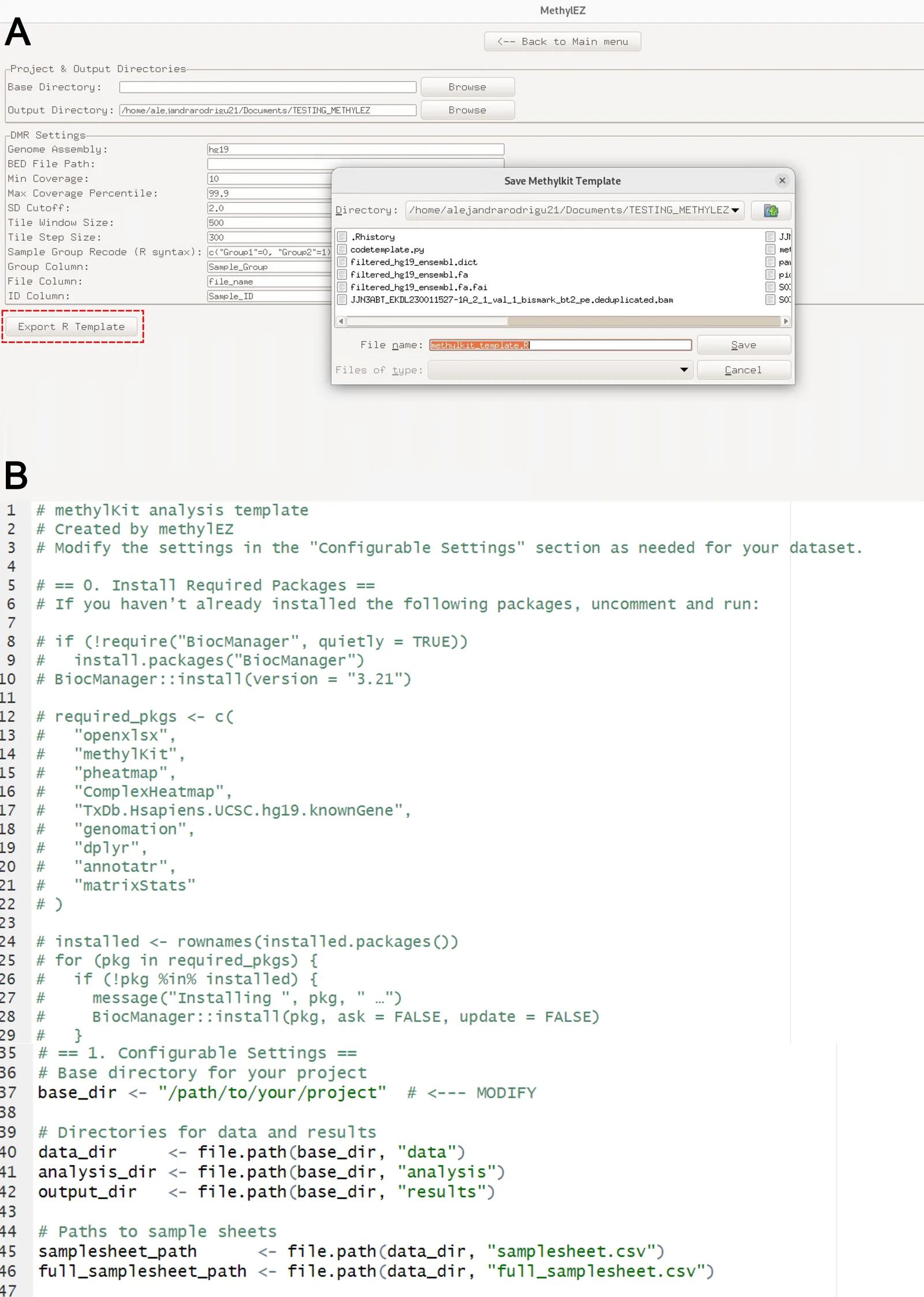
Module 3-methylKit interface and template snippet. (A) User-customizable options: directory where the aligned samples are located (Base Directory), directory for the output (Output Directory) and different methylKit options that can be specified for both differential methylation sites (DMS) and differential methylation regions (DMRs) analyses. Upon selecting Export R Template, the user will be able to save the methylkit template in the output directory selected. (B) Snippet of the first 47 lines of the.R script, depicting the packages that are required and the configurable settings. If the user wants to modify the template directly, the fields are marked with an indicatoron the corresponding line that reads “<-- MODIFY”.

### Validation against public benchmarks

To demonstrate the empirical reliability of *methylEZ*, we processed the public GSE93507 dataset from Kotini et al. (2017) and made the output available in the project’s GitHub repository.

This dataset comprises an Enhanced Reduced Representation Bisulfite Sequencing (ERRBS) experiment profiling DNA methylation in low-risk MDS iPSC-derived haematopoietic progenitor cells (MDS-1.12 line) treated with 5-Azacytidine (5-AzaC), a hypomethylating agent used as first-line therapy in MDS. Reads were aligned to hg19 using Bismark with a 20 Phred quality threshold, and differential methylation was reported using methylKit with thresholds of ≥25% methylation difference and q < 0.01. A samplesheet assigning sample ID, the group (control, high, low) and the file name was absent from the repository. Therefore, we had to create our own as requested by methylKit for further analyses (Table S1).

There are six samples available in the repository corresponding to two controls and four 5-AzaC-treated samples spanning two doses, 100 and 500 nM respectively. As *methylEZ* does not substitute the necessity for interpretation, we had to adjust some of the parameters based on reasonable biological assumptions based on the artcile by Kotini et al. (2017). Pooling the four treated samples into a single group could constitute pseudoreplication, since the two doses represent different experimental conditions and not biological replicates of the same treatment. Since we want to avoid inflation of replicate count and prevent methylKit’s overdispersion-corrected logistic regression to become overconfident, we restricted the primary comparison to high-dose treated *vs*. untreated controls, yielding a 2 *vs*. 2 design.

Coverage files were processed using the *methylEZ* methylKit template, choosing the “custom” pipeline approach. Exact replication of the results from Kotini et al. (2017) was not achievable, owing to several underreported methodological details in the original study. Specifically, the authors do not report their filterByCoverage parameters, whether unite() was run requiring coverage across all samples or with a min.per.group threshold, whether blacklist or repeat-region masking was applied, or the version of methylKit used. This is a common level of methodological underreporting in epigenomics literature.

Nonetheless, our analysis does recover a comparable number of differentially methylated loci to the 30,599 reported at the stated thresholds, plus we achieve a consistent directional profile towards hypomethylation, which does follow the reported findings.

Residual discrepancies in the *p*-value distribution are consistent with the aforementioned parameter uncertainty, and could likely be reduced through systematic parameter sweeping. An exhaustive reproduction of a single published figure is, however, outside the scope of *methylEZ*, especially where there is a consistent lack of methodology as it usually happens. This validation exercise however shows how the tool can produce a useful template that even in partially informed circumstances can provide biologically meaningful, quantitatively comparable output on a well-characterised dataset.

### Comparison with related methylation tools (sequencing, arrays, CLI)

Building on the brief mention of already existing tools in the Introduction section, now we expand on their comparison, which is further explored in Table 2. Methyl-C analyzer (Lu et al., 2023) provides downstream analysis and visualization in methylation but needs pre-processed inputs and a certain scripting familiarity. methylR (Volpe & Das, 2023) simplifies the differential methylation analysis, but it is optimised for Illumina Infinium array data instead of sequencing data. ShinyÉPICo and MethGET (Morante-Palacios & Ballestar, 2021; Teng et al., 2020) also have GUIs but they are designed for microarray data. Other environments like Galaxy (The Galaxy Community, 2024), GATK toolkit (Van der Auwera & O'Connor, 2020) and ENCODE (The ENCODE Project Consortium, 2012) provide a solid integration of complex pipelines, but their main constraint is that they require web-only execution, and they depend on server configurations. BioPipelineCreator (Cordeiro et al., 2024) allows for a graphical setup and management of biological data pipelines, but it is a general-purpose visual builder.

**Table 2:** Feature comparison between *methylEZ* and other GUI or pipeline-based methylation analysis tools. A total of 8 tools, including *methylEZ* describing eight key features of interest: practical category, expected input format, workflow starting point, preprocessing, visualization, reproducibility, extensibility and platform compatibility.

Tool	Expected input format	Workflow starting point	Preprocessing	Visualization	Reproducibility	Extensibility	Platform compatibility
*methylEZ*	Module 1: raw FAST/Q files Module 2: BAM/BAI files Module 3: coverage files	Raw BS-seq FASTQ (Module 1) → QC on aligned outputs (Module 2) → methylKit R template (Module 3).	Yes—automates nf-core/methylseq configuration and QC integration	No in-GUI plots; exports QC metrics (CSV) and an R template that generates plots.	Configurable templates for reproducibility	Expandable with additional analysis modules	Desktop GUI Windows/macOS/Linux; local/offline
MethylC-analyzer	CGmap/CGmap.gz files	Post-alignment methylation analysis and visualization	No—requires pre-aligned and methylation-called data	Static and interactive methylation plots, genome browser support	Standardized output, reproducible scripts	Modular R code	R package + scripts; Windows/macOS/Linux; local
methylR	IDAT files	Microarray methylation data analysis	Yes—tailored to methylation microarray preprocessing	Interactive dashboards and plots	Automated report generation	R Shiny-based, extendable with R code	R Shiny app; Windows/macOS/Linux; local or server-hosted
ShinyÉPICo	IDAT files (arrays)	Array normalization, QC, then DMP and DMR calculation	Yes—minfi normalization, QC metrics, filtering and data exploration	Yes—DMP + DMR *via* mCSEA	Users can export R objects, scripts and HTML reports with the parameters and steps	*Via* R scripts export and Shiny framework	R Shiny app; Windows/macOS/Linux; local or server-hosted
MethGET	CGmap, gene expression table and GTF	Correlation analysis between DNA methylation and gene expression after data preprocessing	Assumes methylation and expression are already processed	Yes—scatterplots, density plots, metagene plots, heatmaps and correlation statistics	Web UI provides downloadable figures. Reproducible execution *via* CLI for saved results.	Configurable analysis parameters *via* GUI/CLI	Web app; optional standalone or CLI (Linux only); browser-based GUI
Galaxy	Dependent on selected workflow (*e.g*., FASTQ, BAM, BED files)	User-defined pipelines for a wide range of omics analyses	Yes (*via* workflows/wrappers installed by admins); supports WGBS.	Built-in interactive plots and third-party tool integration	Workflow histories, version tracking	Large library of tools, community-developed	Web platform (instance-dependent); user OS agnostic; server/admin managed.
BioPipelineCreator	Dependent on selected pipeline	User-defined (graphical pipeline builder).	Possible if user adds steps (not methylation-specific).	None by default; depends on tools users include.	Project configuration export	Flexible pipeline management	Desktop GUI (Java); Windows/macOS/Linux
msPIPE*	**WGBS FASTQ, reference genome, configuration file**	**Raw bisulfite sequencing FASTQ files**	**Yes—trimming, QC, alignment**	**Yes—publication-quality plots**	**Docker containerization ensures reproducibility**	**Configurable R/Perl/Python pipeline**	**CLI (Docker/Conda); Windows/macOS/Linux; no GUI.**

**Note:**

* not a GUI, but a similar workflow to *methylEZ*.

Users compose arbitrary steps and manage tool versions on their own, which can reduce standardization and reproducibility compared with other workflows. Notably remarkable is the tool msPIPE (Kim et al., 2022), which has been included in the comparison because its workflow and goals fully align with that of *methylEZ*, with the main difference being the absence of a UI, being accessible only *via* CLI.

With this in mind, we compiled a comparative table outlining the features of representative platforms across seven different axes: 1. the expected input format, 2. the tool’s workflow starting point, 3. preprocessing capabilities, 4. visualization, 5. reproducibility, 6. extensibility and 7. platform compatibility (Table 2). Tools as the above-mentioned ones were chosen from a range of options as representative solutions with clearly defined target audiences and functionality scopes. They fall into four practical categories. The first one, downstream sequencing GUIs, which are MethylC-analyzer and MethGET, as their starting point is post-alignment summaries. They do not provide raw FASTQ pre-processing. The second category are array-focused GUIs, where we can find methylR and ShiyÉPICo, exclusively working with Illumina array data. The third class corresponds to more general-purpose graphical tools, those that permit for customisable workflows through a web UI or a visual builder, but which are not necessarily methylation specific. Finally, we have a reference workflow category for workflow pipelines that do not have a GUI but that have a similar processing than *methylEZ*, but through a CLI system alone.

## Discussion

*methylEZ* has been developed to address the ongoing challenge of the gap between the flexibility and power of CLI pipelines as well as the accessibility required by researchers with minimal computational expertise. As epigenomics expands into translational and clinical contexts, the need for intuitive, reproducible and customizable tools that contribute to lower the barrier to pipeline use becomes more relevant.

*methylEZ*, operates solely as a configuration GUI, wrapping nf-core/methylseq pipeline together with methylkit R package and some accessory scripts that facilitate running the complete pipeline. It is therefore, not on its nature to output comparable results to other established workflows. Therefore, it strictly generates the commands and templates required to run the nf-core pipeline and R DM analysis, its output is mathematically identical to running those standard pipelines directly through scripting and command line.

The core strength of GUI of *methylEZ* is its modular architecture. By providing straightforward interfaces for preprocessing (sample selection, aligner configuration, command-line generation), quality control (Picard CollectHsMetrics integration) and post-processing sections (R-based DM script generation), this tool enables users to assemble fully reproducible methylation workflows needing just minimal coding. At the same time, it does not constrain the options for each step by focusing on using rigid templates. All exported components, including nf-core commands, Python and R scripts remain fully transparent, portable and most importantly editable, allowing more experienced users to also benefit from them by adapting them as needed. As highlighted in Table 2, amongst all compared tools, *methylEZ* combines template-based command and samplesheet generation, local and offline execution and provides ready-to-submit Nextflow commands for HPC environments. Web-based tools like Galaxy (The Galaxy Community, 2024) do offer very rich, user-friendly user interfaces, however, they typically ask for server/admin configuration in HPC jobs, building on the data sharing considerations. *methylEZ* targets the pre-processing stage, QC and analysis template gap for lab users, while still benefiting from local control.

A major differentiating feature of *methylEZ* is its support for pre-processing, a stage that is often overlooked. Most existing tools will require that the user provides already-aligned or methylation-called data as input. As outlined in the comparative table, while Galaxy technically allows for pre-processing, through its community-developed plugins, however its implementation relies heavily on wrappers of external tools, which require access to a server with the tools pre-installed or to administrative privileges to add them. Moreover, users are dependent on the versions and configurations available within that specific Galaxy instance, which may include outdated packages or non-debugged versions. This setup introduces potential reproducibility issues and there is an inherent loss of transparency due to the lack of replication of these steps.

Aside from GUI-oriented tools, the role of established CLI pipelines and community frameworks for methylation analysis standardization must be considered. As I have previously mentioned, the tool msPIPE (Kim et al., 2022) represents a comprehensive WGBS pipeline which starts with FASTQ data and runs a whole workflow from alignment to visualization. Although it provides end-to-end reproducibility and robust functionality, the absence of a GUI implementation makes it less accessible to any researcher lacking computational training. Similarly, the ENCODE Project (Van der Auwera & O'Connor, 2020; The ENCODE Project Consortium, 2012) has defined gold-standard workflows for epigenomic assays, but, like msPIPE, does not provide a GUI as it is typically run on cloud or cluster infrastructures.

On the other hand, BioPipelineCreator (Cordeiro et al., 2024) includes a more hands-on approach, allowing users to visually assemble pre-processing pipelines that include steps such as FASTQ trimming, mapping, and sorting, conceptually similar to the structure employed by nf-core/methylseq. However, the flexibility it offers does nonetheless come at the cost of standardization and can hinder reproducibility, particularly when there is an associated heterogeneity to the tools used which are not maintained under one unified framework. To overcome this limitation, *methylEZ* prioritizes the use of community-validated workflows such as nf-core/methylseq, ensuring both standardization and reproducibility while minimizing user setup complexity.

As previously outlined, all the reviewed tools present unique paradigms in their design, some prioritising full specialization for certain data types, others prioritising the flexibility of the environment itself. In this context, *methylEZ* emphasizes template generation and local control. Although other platforms offer methylation workflows through graphical configuration, they typically require server infrastructure and provide limited support for customization. In contrast, *methylEZ* assumes that users will execute workflows on local machines or HPC environments, offering greater flexibility and control.

An additional advantage of *methylEZ* is its adaptability across different operating systems and its minimal dependency requirements. The tool is easily installable *via* pip, requires just standard Python libraries, a few well supported packages like pandas or pyperclip. A bundled picard.jar file is provided to reduce setup complexity. Users will need to ensure that Java is on PATH for it to work. The current framework was designed with extensibility in mind. The next planned feature is the addition of a dedicated visualization module, which will allow users to explore QC metrics and differential methylation results directly within the GUI, including plots like PCA, coverage histograms and volcano plots, which are already considered, but only get generated where the R file is run. This would complete the analytical loop, providing a unified graphical environment.

There is a specific room for lightweight GUI tools, for example, re-Searcher (Karabayev et al., 2021), which offers simplified interfaces for tasks like variant filtering and VCF exploration. *methylEZ* expands upon this genre by integrating GUI-based interactions with entire analysis stages ranging from raw input pre-processing to downstream differential methylation testing.

Although current limitations include the lack of direct visualization of plots embedded in the GUI and being able to run a differential methylation analysis in the GUI as well, the core functionality has been implemented in a system independent fashion. Future versions will focus on expanding the software’s capabilities with integrated visualization modules and support for alternative methylation callers and statistical frameworks.

In summary, *methylEZ* is user-oriented, reproducible and modular. It provides a solution for navigating the complexity of methylation sequencing analysis and complements existing tools, focusing on bridging the gap between CLI power and GUI usability, positioning itself as a scalable foundation for further community-oriented development.

### Limitations

While *methylEZ* significantly streamlines the setup and execution of bisulfite sequencing workflows, several limitations must be transparently acknowledged.

#### Computational resource requirements

Despite providing a simplified graphical interface, *methylEZ* still requires substantial computational infrastructure. Because the underlying aligners (Bismark, bwa-meth) are computationally intensive, truly “local” execution is strictly limited to high-performance workstations or HPC environments, rather than standard laptops. For example, Bismark typically requires 8–12 GB of RAM for mammalian genomes, alignments can take 24–48 h per sample on standard multi-core workstations, and whole-genome datasets generate large intermediate BAM files often exceeding 100 GB per study.

#### Pipeline dependencies

*methylEZ* enables reproducible analysis by leveraging nf-core/methylseq; however, this creates inherent dependency risks. The tool’s functionality relies on the maintenance of the nf-core pipeline, which is subject to version changes, algorithm updates, and shifting default parameters. Furthermore, nested dependencies (*e.g*., Bismark, BWA, Samtools) introduce their own version management challenges.

#### Dependency on upstream QC

*methylEZ’*s output quality is fundamentally constrained by the QC and filtering performed by nf-core/methylseq. While nf-core provides conversion rate metrics, *methylEZ* does not independently validate conversion efficiency. Users working with poorly converted samples may obtain unreliable methylation estimates and must inspect Bismark or MultiQC reports independently.

#### Limited downstream filtering automation

*methylEZ* does not currently implement post-calling automated filters. Users performing differential methylation analysis must manually apply experimental-specific filters, such as coverage-based CpG filtering (minimum coverage thresholds or removal of high-coverage outliers suggesting PCR bias), standard deviation filtering for non-variable CpGs, and SNP-aware filtering to remove probes overlapping common variants.

## Conclusions

*methylEZ* was developed to address a defined gap in the DNA methylation analysis ecosystem defined by the lack of a modular, GUI-based tool for preparing and organizing BS-seq workflows. The tool provides a completely graphical interface for configuring nf-core/methylseq pipelines, quality control metrics, and exporting customizable R scripts for differential methylation analyses. These features collectively reduce the entry barrier for lab-based researchers or researchers with minimal scripting expertise but remaining useful by being completely editable for experienced users. The most significant contribution of *methylEZ* lies in its capacity to serve as a lightweight, locally executable assistant that generates standardized, reproducible scripts across key stages of methylation analysis. By focusing on configuration and template export rather than execution, it offers flexibility across computational environments, particularly for high-performance computing and containerized workflows.

Ultimately, *methylEZ* contributes to the ongoing effort to make epigenomic analysis more reproducible, accessible, and customizable, which is openly available to the community and is expected to evolve alongside emerging user requirements.

## Supplemental Information

10.7717/peerj.21795/supp-1 Supplemental Information 1 Sample file used to test the methylEZ modules 1 and 2. This file alongside with Ecoli_10K_methylated_R2.fastq.gz are part of what has been used to test the functionalities of modules 1 and 2 in *methylEZ*. This is a paired-end read sample.

10.7717/peerj.21795/supp-2 Supplemental Information 2 Second sample file used to test the *methylEZ* modules 1 and 2. This file alongside with Ecoli_10K_methylated_R1.fastq.gz are part of what has been used to test the functionalities of modules 1 and 2 in *methylEZ*. This is a paired-end read sample.

10.7717/peerj.21795/supp-3 Supplemental Information 3 Samplesheet generated to process **GSE93507** data with *methylEZ*. Sample_ID corresponds to the ID assigned to each sample according to the raw data downloaded from GEO. Sample_Group column indicates if the sample corresponds to a control sample (Control), to a 100 nM 5-AzaC incubated (Low) or to a 500 nM 5-AzaC incubated (High) sample. file_name column indicates the name of the un-tar files as extracted from the GEO download. Title column provides extra information according to the artcile associated to the experiment.

10.7717/peerj.21795/supp-4 Supplemental Information 4 Sample used to test functionalities for single-end reads (1 out of 3). This file alongside with SRR389222_sub#.fastq.gz are part of what has been used to test the functionalities of modules 1 and 2 in *methylEZ*. This is one of three single-end read samples.

10.7717/peerj.21795/supp-5 Supplemental Information 5 Sample used to test functionalities for single-end reads (2 out of 3). This file alongside with SRR389222_sub#.fastq.gz are part of what has been used to test the functionalities of modules 1 and 2 in *methylEZ*. This is one of three single-end read samples.

10.7717/peerj.21795/supp-6 Supplemental Information 6 Sample used to test functionalities for single-end reads (3 out of 3). This file alongside with SRR389222_sub#.fastq.gz are part of what has been used to test the functionalities of modules 1 and 2 in *methylEZ*. This is one of three single-end read samples.

## Additional Information and Declarations

### Data Availability

The following information was supplied regarding data availability:

The data is available in the Supplemental Files.

The code is available at GitHub and Zenodo:

- https://github.com/AlejRSosa/methylEZ/.

- Alejandra Rodriguez-Sosa. (2026). AlejRSosa/*methylEZ*: methylEZ_v_0.1 (Version v_0.1) [Computer software]. Zenodo. https://doi.org/10.5281/zenodo.20023775.

The validation data is available at NCBI GEO: GSE93507.
